# Metastatic adenocarcinoma after laparoscopic supracervical hysterectomy with morcellation: A case report^☆^

**DOI:** 10.1016/j.gynor.2013.03.002

**Published:** 2013-03-16

**Authors:** Taylor Turner, Angeles Alvarez Secord, William J. Lowery, Gregory Sfakianos, Paula S. Lee

**Affiliations:** a Department of Obstetrics and Gynecology, Duke University Medical Center, USA; b Division of Gynecologic Oncology, Department of Obstetrics and Gynecology, Duke University Medical Center, USA; c Duke Cancer Institute, USA

**Keywords:** Uterine morcellation, Laparoscopic hysterectomy, Disseminated malignancy

## Abstract

• Uterine morcellation is common in minimally invasive hysterectomy but should be performed with caution due to risk of unsuspected malignancy.
• Intraoperative techniques should be considered to minimize dissemination of endometrial tissue during morcellation.
• Strategies to ensure accurate pathologic evaluation of morcellated specimens and to improve preoperative risk stratification before morcellation procedures are necessary.

## Introduction

Since its introduction in the mid 1990s mechanical intra-abdominal morcellation of uterine tissue at the time of laparoscopic surgery is commonly performed to facilitate the removal of large uteri or leiomyomas through small incisions. However, with mechanical morcellation there are reported risks of metastasizing leiomyosarcoma, uterine cancer, introduction of complications from retained fragments of myomas, and endometriosis (Lieng et al., 2006; Sepilian and Della Badia, 2003). Here we present a case that further illustrates the potential malignant risk of uterine morcellation and the uncertainty regarding the primary site of tumor origin.

## Case report

A perimenopausal 56 year old nulliparous woman underwent supracervical laparoscopic hysterectomy and bilateral salpingoophorectomy for pelvic pain, menorrhagia, and large uterine leiomyomas. She had no history of abnormal Pap smears and a preoperative Pap smear was normal. Her family history was negative for any known gynecologic, breast, or gastrointestinal malignancies. Preoperative chest radiograph and endometrial biopsy were not obtained. Due to menorrhagia, an endometrial curettage was performed at the initiation of the procedure. The specimen was sent for frozen section and was negative for malignancy. Intraoperative findings were significant for an 18–20 week size uterus weighing 1518 g. Prolonged mechanical morcellation with a ROTOCUT G1 morcellator (Karl Storz, Germany) was required to remove the uterus and a small amount of superficial endometriosis in the pelvis was seen and cauterized. The fallopian tubes and ovaries were removed intact in an endoscopic bag. The uterus was not morcellated in a bag, and the fragments were subsequently collected from within the abdomen, and withdrawn from a laparoscopic port. Final pathology on six uterine fragments revealed weakly proliferative phase endometrium without atypia, adenomyosis and leiomyoma in the uterus. The ovaries showed fragments of benign ovarian parenchyma and patchy endosalpingiosis. No malignancy was identified in any specimen.

**Fig. 1:**
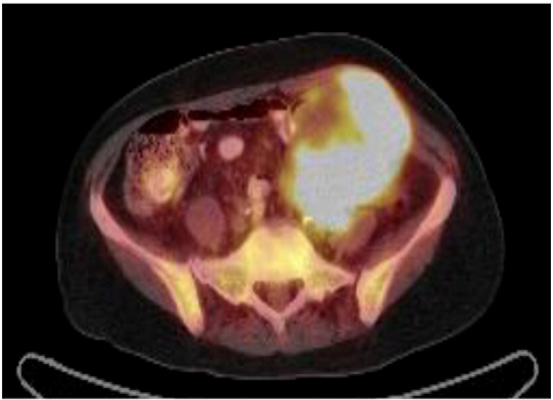
Fused PET/CT coronal image of the large hypermetabolic pelvic mass.

**Fig. 2:**
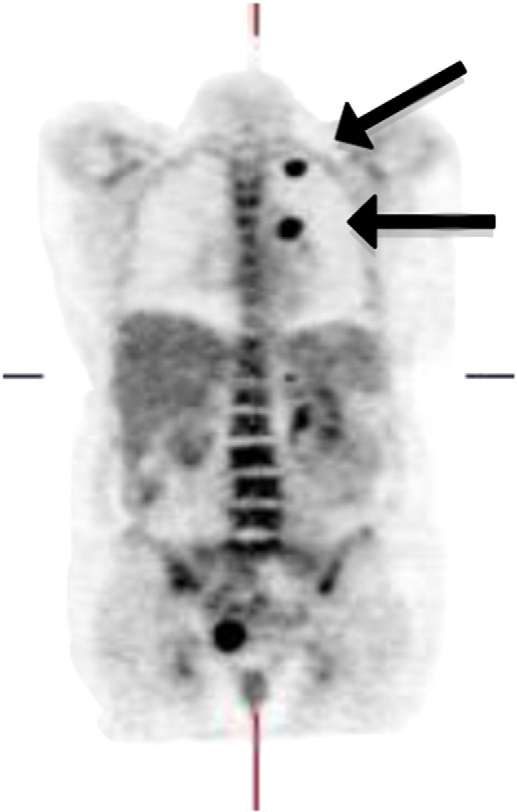
Fused PET/CT sagittal section showing two hypermetabolic lung nodules (marked with arrows).

The patient's initial recovery was unremarkable until 14 months post-operatively when she presented with left lower quadrant pain and persistent cough with shortness of breath. Examination revealed a firm palpable abdominal mass at the left lower quadrant and a fleshy lesion at the apex of the vagina. The cervix could not be identified. PET–CT revealed multiple hypermetabolic masses in the pelvis (Fig. 1), abdomen, and chest (Fig. 2) concerning for metastatic disease. A core needle biopsy from the 9 cm left pelvic mass revealed poorly differentiated malignant neoplasm of epithelioid cells with hyperchromatic nuclei in a background of necrosis. Immunohistochemical stains showed tumor cells positive for cytokeratin, vimentin, and CD138. The tumor cells were negative for SMA, EMA, ER, TLE1, HNB45, desmin, myogenin, CD17, BCL2, inhibin, MDN2, CD4, CA125, WT-1, CD45, calretinin, DOG-1, p16, CEA, CK 20, HPV, and CD99. The vaginal biopsy showed grade 3 endometrioid adenocarcinoma. Pathology re-review of the supracervical hysterectomy and bilateral salpingo-oophorectomy specimens showed no evidence of malignancy. The differential diagnosis included recurrent uterine cancer (unrecognized at time of morcellation) versus primary peritoneal carcinoma that developed from endometriosis.

The patient was treated with intravenous carboplatin AUC 6 and paclitaxel 175 mg/m^2^ every 21 days and had a partial response after two cycles. She completed six cycles of therapy, but unfortunately developed rapidly progressive disease. One month after her sixth cycle of chemotherapy, she presented with a complex fistula involving the pelvic tumor, abdominal wall, and sigmoid colon. She underwent a diverting loop colostomy. She strongly desired further therapy and received one cycle of doxorubicin 60 mg/m^2^. After admission for febrile neutropenia and pulmonary embolism, she elected to transition her care to hospice and died of her disease two months later.

## Discussion

Our case illustrates the potential risk of metastatic adenocarcinoma diagnosed after hysterectomy with uterine morcellation. Definite causal relationship of metastatic disease as a result of morcellation cannot be proven; however, in the setting of an unknown cancer diagnosis at time of morcellation, subsequent widespread dissemination in the pelvis may result. Others have reported metastatic leiomyosarcoma, disseminated endometriosis (Sepilian and Della Badia, 2003), pelvic atypical endometrial hyperplasia (Kill et al., 2011), and uterine cancer after uterine morcellation (Einstein et al., 2008). In the largest case–control study to date, morcellation of unsuspected leiomyosarcoma was associated with an increased risk of peritoneal recurrence and worse overall survival (Park et al., 2011).

In our case the primary site of malignancy was uncertain. A plausible source could be that she had an unrecognized malignancy at the time of initial hysterectomy. Schneider et al. reported a case of undifferentiated adenocarcinoma five months after laparoscopic supracervical hysterectomy. The patient had undergone a macromorcellation supracervical hysterectomy and vaginal intrafascial cylindriform enucleation of the cervix and corpus uteri. In this procedure, the first core is performed vaginally to allow for a contiguous punched out specimen containing the cervix, endocervix, endometrium, and myometrium to optimize pathological evaluation. However, even with this additional step, initial pathology review showed no evidence of malignancy. When she was diagnosed with undifferentiated adenocarcinoma of a pelvic mass, retrospective review of the initial morcellated hysterectomy revealed clusters of malignant cells attached to the endocervical epithelium. However, the definite source of the malignant tissue could not be identified (Schneider, 1997). Although our case did not show evidence of malignancy at the time of hysterectomy, it is plausible that the malignancy was unrecognized. Two considerable diagnostic challenges were the large uterine size and the morcellation. Rivard and colleagues demonstrated the challenges of accurate detection of endometrial cancer in morcellated specimens. In this prospective case series, after standard processing and diagnosis of endometrial disease from intact uteri, all specimens were then morcellated. A single pathologist blinded to the initial diagnosis reviewed each morcellated specimen. The diagnosis of malignancy was missed in 4 of 5 specimens of known cancer (Rivard et al., 2012).

Another possible source of the malignancy is the malignant transformation of retained endometrial tissue or endometriosis after morcellation. Our patient had endometriosis involving the serosa of the uterus. Interestingly, Schuster et al. compared morcellated to non-morcellated hysterectomy cases and found no significant difference in outcomes in women that had endometriosis diagnosed at the time of hysterectomy. In this case–control study of 464 cases there was no difference in the incidence of new onset endometriosis between women undergoing a supracervical hysterectomy with uterine morcellation versus those women undergoing traditional vaginal or abdominal hysterectomy without morcellation (Schuster et al., 2012). However, reoperation for those with endometriosis in the morcellation group did show persistent or recurrent endometriosis. Endometriosis is associated with an increased risk of endometrioid invasive ovarian cancer (Pearce et al., 2012). Further investigation is needed to understand the risk factors of malignant transformation of endometriosis.

Minimally invasive surgery (MIS) has well documented benefits and uterine morcellation plays an important role in facilitating less invasive procedures for large uteri. However, the potential morbidity from mechanical uterine morcellation must be recognized. Appropriate pre-operative screening to rule out endometrial carcinoma in asymptomatic women prior to morcellation is challenging. Of the 708 women undergoing hysterectomy for pelvic organ prolapse, the incidence of endometrial cancer was only 0.6% (Ramm et al., 2012). Furthermore, 4 out of the five cancers detected in this study had normal preoperative screening with endometrial biopsy, ultrasound, or both tests. Thus, routine preoperative evaluation of asymptomatic women did not yield additional benefit and may be cost prohibitive (Ramm et al., 2012). Intraoperative attention to removal of all fragments of tissue at time of morcellation is imperative. In addition alternative morcellation techniques such as vaginal morcellation in a sterile bag, controlled abdominal port morcellation in a sterile bag, or minilaparotomy after MIS to remove the specimen intact to avoid dissemination of endometrial tissue into the abdominal cavity should be considered. Potential strategies need to be developed to improve accuracy of pathologic evaluation of morcellated specimens. Because malignant complications from morcellation are rare, conclusive statements cannot be made on the causality of morcellation to subsequent development of malignancy. Nevertheless, given the increasing case reports of poor outcomes after morcellation, further investigation in developing risk stratification to identify women who should avoid a morcellation procedure should be considered.

## Conflict of interest statement

None of the authors have any potential conflicts of interest.
